# TRPM2 channel deficiency prevents delayed cytosolic Zn^2+^ accumulation and CA1 pyramidal neuronal death after transient global ischemia

**DOI:** 10.1038/cddis.2014.494

**Published:** 2014-11-27

**Authors:** M Ye, W Yang, J F Ainscough, X-P Hu, X Li, A Sedo, X-H Zhang, X Zhang, Z Chen, X-M Li, D J Beech, A Sivaprasadarao, J-H Luo, L-H Jiang

**Affiliations:** 1 Department fof Neurobiology, Institute of Neuroscience, Key Laboratory of Medical Neurobiology of the Ministry of Health of China, Zhejiang Province Key Laboratory of Neurobiology, Zhejiang University School of Medicine, Hangzhou, Zhejiang 310058, China; 2 School of Biomedical Sciences, Faculty of Biological Sciences, University of Leeds, Leeds LS2 9JT, UK; 3 School of Medicine, Faculty of Health and Medicine, University of Leeds, Leeds LS2 9JT, UK; 4 Department of Physiology and Neurobiology, Xinxiang Medical University, Henan 453003, China

## Abstract

Transient ischemia is a leading cause of cognitive dysfunction. Postischemic ROS generation and an increase in the cytosolic Zn^2+^ level ([Zn^2+^]_c_) are critical in delayed CA1 pyramidal neuronal death, but the underlying mechanisms are not fully understood. Here we investigated the role of ROS-sensitive TRPM2 (transient receptor potential melastatin-related 2) channel. Using *in vivo* and *in vitro* models of ischemia–reperfusion, we showed that genetic knockout of TRPM2 strongly prohibited the delayed increase in the [Zn^2+^]_c_, ROS generation, CA1 pyramidal neuronal death and postischemic memory impairment. Time-lapse imaging revealed that TRPM2 deficiency had no effect on the ischemia-induced increase in the [Zn^2+^]_c_ but abolished the cytosolic Zn^2+^ accumulation during reperfusion as well as ROS-elicited increases in the [Zn^2+^]_c_. These results provide the first evidence to show a critical role for TRPM2 channel activation during reperfusion in the delayed increase in the [Zn^2+^]_c_ and CA1 pyramidal neuronal death and identify TRPM2 as a key molecule signaling ROS generation to postischemic brain injury.

Transient ischemia is a major cause of chronic neurological disabilities including memory impairment and cognitive dysfunctions in stroke survivors.^1, 2^ The underlying mechanisms are complicated and multiple, and remain not fully understood.^3^ It is well documented in rodents, non-human primates and humans that pyramidal neurons in the CA1 region of the hippocampus are particularly vulnerable and these neurons are demised after transient ischemia, commonly referred to as the delayed neuronal death.^4^ Studies using *in vitro* and *in vivo* models of transient ischemia have demonstrated that an increase in the [Zn^2+^]_c_ or cytosolic Zn^2+^ accumulation is a critical factor.^5, 6, 7, 8, 9, 10, 11^ There is evidence supporting a role for ischemia-evoked release of vesicular Zn^2+^ at glutamatergic presynaptic terminals and subsequent entry into postsynaptic neurons via GluA2-lacking AMPA subtype glutamate receptors (AMPARs) to raise the [Zn^2+^]_c._^12, 13, 14, 15, 16^ Upon reperfusion, while glutamate release returns to the preischemia level,^17^ Zn^2+^ can activate diverse ROS-generating machineries to generate excessive ROS as oxygen becomes available, which in turn elicits further Zn^2+^ accumulation during reperfusion.^18, 19^ ROS generation and cytosolic Zn^2+^ accumulation have a critical role in driving delayed CA1 pyramidal neuronal death,^7, 12, 20, 21, 22^ but the molecular mechanisms underlying such a vicious positive feedback during reperfusion remain poorly understood.

Transient receptor potential melastatin-related 2 (TRPM2) forms non-selective cationic channels; their sensitivity to activation by ROS via a mechanism generating the channel activator ADP-ribose (ADPR) confers diverse cell types including hippocampal neurons with susceptibility to ROS-induced cell death, and thus TRPM2 acts as an important signaling molecule mediating ROS-induced adversities such as neurodegeneration.^23, 24, 25, 26^ Emergent evidence indeed supports the involvement of TRPM2 in transient ischemia-induced CA1 pyramidal neuronal death.^27, 28, 29, 30^ This has been attributed to the modulation of NMDA receptor-mediated signaling; despite that ROS-induced activation of the TRPM2 channels results in no change in the excitability of neurons from the wild-type (WT) mice, TRPM2 deficiency appeared to favor prosurvival synaptic Glu2A expression and inhibit prodeath extrasynaptic GluN2B expression.^30^ A recent study suggests that TRPM2 activation results in extracellular Zn^2+^ influx to elevate the [Zn^2+^]_c_.^31^ The present study, using TRPM2-deficient mice in conjunction with *in vivo* and *in vitro* models of transient global ischemia, provides compelling evidence to show ROS-induced TRPM2 activation during reperfusion as a crucial mechanism determining the delayed cytosolic Zn^2+^ accumulation, CA1 neuronal death and postischemic memory impairment.

## Results

### TRPM2 channels are functionally expressed in hippocampal pyramidal neurons

**Figure 1:**
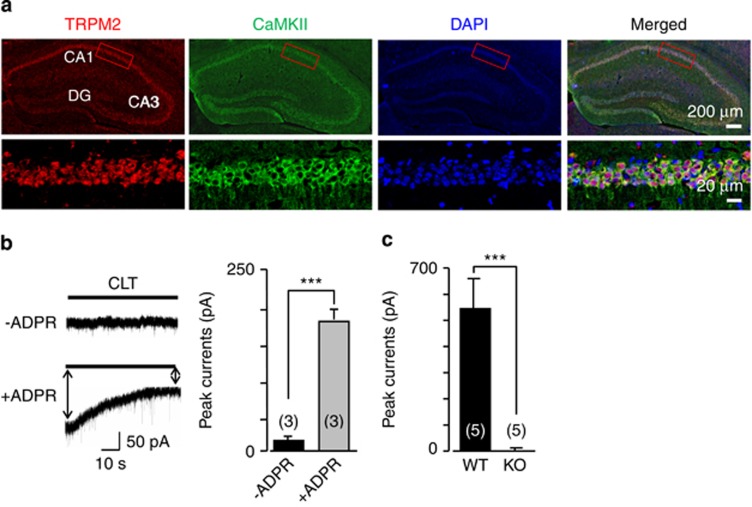
TRPM2 expression in hippocampal pyramidal neurons. (**a**) Representative immunofluorescent images showing expression of TRPM2 in CaMKII-positive pyramidal neurons in the hippocampus including CA1 region. DAPI (4',6-diamidino-2-phenylindole) staining and merged images are also shown. (**b**) Representative traces showing 1 mM ADPR-induced inward current in WT hippocampal brain slices that was inhibited by 20 *μ*M clotrimazole (CLT). The solid line above the current recording denotes the zero current level. The holding membrane potential was −60 mV. Summary of the currents recorded using intracellular solutions with or without ADPR are shown on the right. (**c**) Mean inward currents induced by 1 mM ADPR in cultured WT or TRPM2-KO hippocampal neurons. The holding membrane potential was −60 mV. The number of neurons recorded for each case is shown within parentheses. ***P<0.005 for comparison of indicated groups

We first examined TRPM2 expression by immunofluorescent staining of hippocampal brain slices and neuron cultures. Strong TRPM2 immunostaining was present in hippocampal neurons (Supplementary Figure 1), particularly in pyramidal neurons identified by the expression of calmodulin-dependent kinase II (CaMKII) (Figure 1a). Whole-cell patch-clamp recordings of CA1 pyramidal neurons in hippocampus slices documented ADPR-induced inward currents, which were inhibited by clotrimazole, a TRPM2 channel inhibitor^23^ (Figure 1b). ADPR-induced currents were also observed in the cultured WT hippocampal neurons, as reported by a previous study,^32^ but were absent in the TRPM2-knockout (KO) hippocampal neurons (Figure 1c). These results are consistent in confirming the functional TRPM2 channel expression in CA1 pyramidal neurons.^32^

### TRPM2 deficiency prevents CA1 pyramidal neuronal death induced by transient ischemia

**Figure 2:**
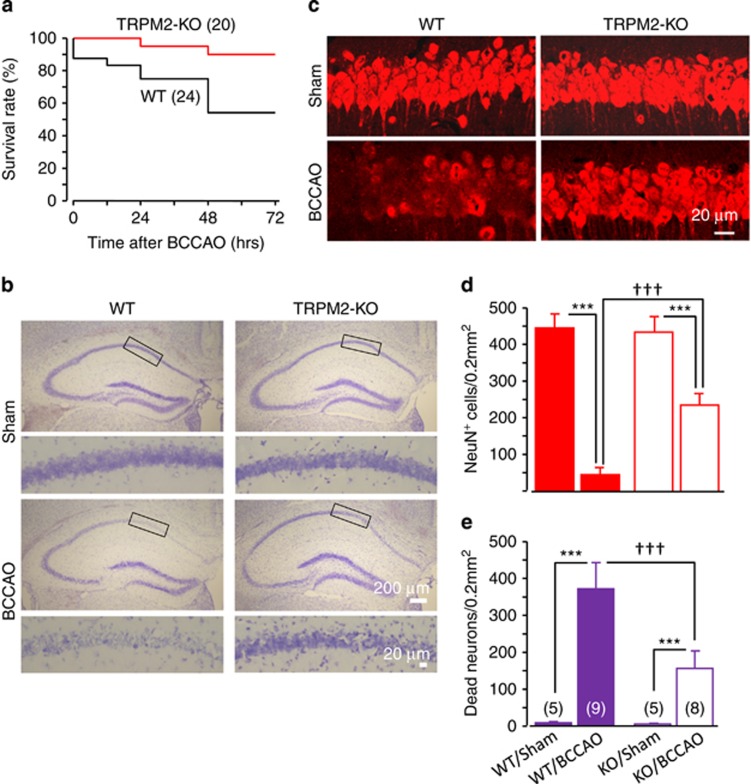
TRPM2 deficiency protects delayed CA1 pyramidal neuronal death induced by transient global ischemia. (**a**) Summary of the survival rate for WT and TRPM2-KO mice during 72 h after BCCAO operation. The number of mice used for each case is shown within parentheses. (**b** and **c**) Representative images showing Nissl (**b**) or NeuN (**b**) staining of hippocampal slices. The areas in the rectangle boxes are shown in enlarged images underneath. (**d** and **e**) Quantitative analysis of NeuN-positive (**d**) and damaged neurons (**e**) in BCCAO- or sham-operated WT and TRPM2-KO mice as shown in (**b** and **c**). The number of mice used for each case is shown within parentheses in (**e**). ***P<0.005 for comparisons within WT or TRPM2-KO mice; ^†††^*P*<0.005 for comparison between WT and TRPM2-KO mice

TRPM2 deficiency caused no discernible effect on the number of hippocampal neurons and GFAP-positive glial cells during development (Supplementary Figures 2a–c). In addition, there was no significant difference in the body weight of adult WT and TRPM2-KO mice. The survival rate of WT and TRPM2-KO mice was examined over a period of 72 h after being subjected to bilateral common carotid artery occlusion (BCCAO) followed by reperfusion, an *in vivo* model of transient global ischemia. The survival rate was 54% for the BCCAO-operated WT mice, which was significantly lower than the survival rate of 90% observed in BCCAO-operated TRPM2-KO mice (*P*<0.05) (Figure 2a). To examine the contribution of TRPM2 in delayed CA1 pyramidal neuronal death, we analyzed the neuronal loss in hippocampal slices from the mice survived 72 h after BCCAO operation (Figures 2b–e). Nissl staining disclosed a huge number of damaged or dead pyramidal neurons in the CA1 region in the BCCAO-operated WT mice (373±72/0.2 mm^2^, *n*=8), but very few damaged neurons in the sham-operated WT mice (9.0±3.5, *n*=5) (Figures 2b and e). The number of damaged neurons was markedly reduced in the BCCAO-operated TRPM2-KO mice (156±48, *n*=9) (Figures 2b and e). Conversely, NeuN staining revealed many more CA1 pyramidal neurons in the TRPM2-KO mice (236±31/0.2 mm^2^, *n*=9) than in the WT mice after BCCAO operation (46±20, *n*=8) (Figures 2c and d). These results clearly demonstrate that TRPM2 has an important role in mediating transient ischemia-induced delayed neuronal death.

### TRPM2 deficiency protects memory impairment after transient ischemia

**Figure 3:**
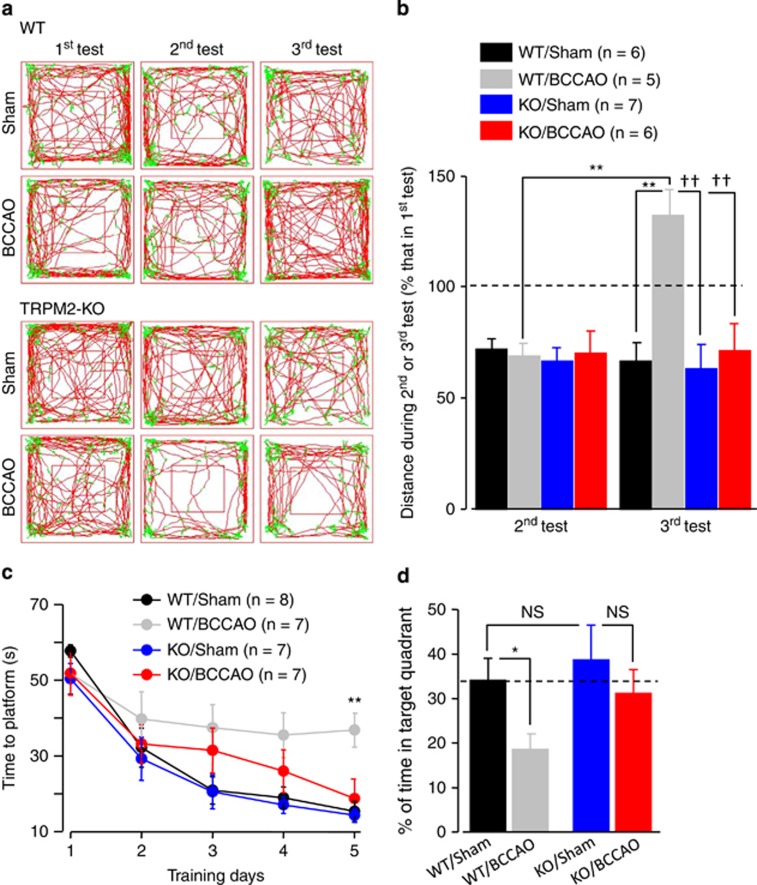
TRPM2 deficiency prevents memory impairment induced by transient global ischemia. (**a**) Representative tracking traces in novelty environment habituation test. BCCAO or sham operation was introduced after the second test. (**b**) Summary of the locomotor activities as shown in (**a**). **P<0.01 for comparison between the activities during the second test before BCCAO and third test after BCCAO in WT mice and also between the activities in the third test of the BCCAO- and sham-operated WT mice. ^††^*P*<0.01 for comparisons between BCCAO-operated WT mice and sham- or BCCAO-operated TRPM2-KO mice. (**c**) Summary of the latency to find the escape platform during the 5-day training session. The number of mice examined for each case is shown within parentheses. ^††^*P*<0.01 for comparisons between BCCAO-operated WT mice and other three groups of mice. (**d**) Summary of the percentage of time searching the escape platform in the target quadrant during the probe test. *P<0.05 for comparison between sham- and BCCAO-operated WT mice and NS, no significant difference for indicated comparisons. The number of mice examined for each case is shown within parentheses in (**b**) for novelty environment habituation test and in (**c**) for water maze test

It is well established that the CA region in the hippocampus is pivotal for learning and memory. We thus evaluated whether TRPM2 deficiency can also protect against transient ischemia-induced memory impairment. The WT and TRPM2-KO mice had similar locomotor function and exploratory ability as assessed by the open-field test (Supplementary Figure 3). However, the WT and TRPM2-KO mice after BCCAO exhibited significant difference revealed by the novelty environment habituation test (Figures 3a and b). Unlike the sham-operated WT mice that showed reduced activities in exploring the pre-exposed environment (66.4±8.3% of the first time, *n*=6), the BCCAO-operated WT mice exhibited significantly increased activities (131.9±11.6%, *n*=5; not significantly different from the activities during the first test), indicating memory impairment (Figure 3b). In striking contrast, the TRPM2-KO mice displayed similar and reduced activities after BCCAO (71.1±12.2%, *n*=6) or sham operation (63.0±10.9%, *n*=7) (Figure 3b).

We also examined the spatial memory with the water maze test (Figures 3c and d). Following a 5-day training session, the latency to find the escape platform was progressively shortened for both WT and TRPM2-KO mice. At the end of the training session, the latency was however significantly longer for the BCCAO-operated WT mice (36.8±4.5 s, *n*=7) than the sham-operated WT (15.3±2.7 s, *n*=8; *P*<0.01) (Figure 3c), confirming impaired learning ability. In contrast, there was no difference in the latency for the BCCAO-operated (18.7±5.2 s, *n*=7) and sham-operated TRPM2-KO mice (14.3±1.8 s, *n*=7) (Figure 3c), indicating that TRPM2 deficiency prevented such learning impairment. During the probe test in which the escape platform was removed, the time the mice used to search for the missing platform in the target quadrant was recorded to determine the spatial memory (Figure 3d). The BCCAO-operated WT mice spent significantly less time in the targeted quadrant (18.6±3.5%, *n*=7) in comparison with the sham-operated WT mice (34.1±5.0%, *n*=8; *P*<0.05), suggesting impaired spatial memory as reported by previous studies.^33^ However, there was no significant difference in the time the TRPM2-KO mice spent in the targeted quadrant after sham (38.7±7.8%, *n*=7) and BCCAO operation (31.2±5.3%, *n*=7) (Figure 3d).

Taken together, these *in vivo* studies provide strong evidence to support a crucial role for TRPM2 in mediating memory impairment after transient ischemia.

### TRPM2 deficiency prevents delayed increase in the [Zn^2+^]_c_

**Figure 4:**
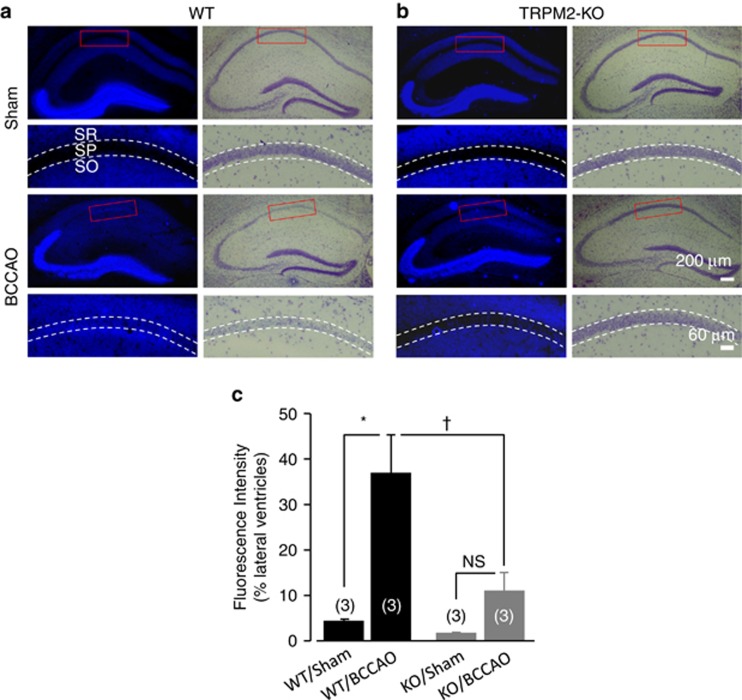
TRPM2 deficiency prevents delayed increases in the [Zn^2+^]_c_ and CA1 pyramidal neuronal death after transient global ischemia. (**a** and **b**) Representative images of TSQ (blue) and Nissl staining of hippocampal brain slices from BCCAO- or sham-operated WT (**a**) and TRPM2-KO mice (**b**). SR, SP and SO represent the stratum radiatum, pyramidale and oriens layers. The areas in the red rectangle box are shown in enlarged images underneath, with the double-dotted lines highlighting the SP layer. (**c**) Summary of the TSQ staining presented as percentage of that in the lateral ventricle as shown in (**a** and **b**). The number of mice examined for each case is shown within parentheses in (**c**). *P<0.05 and NS, no significant difference, for comparisons within WT or TRPM2-KO mice; ^†^*P*<0.05 for comparison between BCCAO-operated WT and TRPM2-KO mice

As mentioned above, there is strong evidence to support the delayed increase in the [Zn^2+^]_c_ as a key factor driving CA1 neuronal death.^3, 5, 9, 10, 11, 14, 34, 35, 36, 37^ Moreover, using single-cell imaging and tetracycline (Tet)-inducible TRPM2-stable HEK293 cells, we found that H_2_O_2_ evoked a modest but significant increase in the [Zn^2+^]_c_ in extracellular Zn^2+^-free solutions and a further >5-fold increase upon introduction to extracellular Zn^2+^-containing solutions in TRPM2-expressing cells (Tet^+^), and, in contrast, there was virtually no H_2_O_2_-induced increase in the [Zn^2+^]_c_ in uninduced cells (Tet^−^) (Supplementary Figure 4). These results are consistent with a recent study that suggests that TRPM2 channel activation results in increases in the [Zn^2+^]_c_.^38^ We therefore hypothesized that TRPM2 deficiency protects the delayed neuronal death by preventing TRPM2-dependent increases in the [Zn^2+^]_c_ during reperfusion. To test this hypothesis, we performed Nissl and TSQ double staining of hippocampal slices from the WT and TRPM2-KO mice 72 h after BCCAO operation to determine postischemic [Zn^2+^]_c_ and cell death in CA1 pyramidal neurons (Figure 4). Neuronal death in the SP layer was clearly accompanied by an increase in the [Zn^2+^]_c_ in the BCCAO-operated WT mice (Figure 4a), and such increases in neuronal death and the [Zn^2+^]_c_ were nearly abolished in the BCCAO-operated TRPM2-KO mice (Figure 4b). Further detailed analysis indicates that the [Zn^2+^]_c_ was significantly increased in the BCCAO-operated WT mice (37.2±8.0, *n*=3) compared with the sham-operated WT mice (4.3±0.5, *n*=3) (Figure 4c). Such an increase was remarkably suppressed in the BCCAO-operated TRPM2-KO mice (11.1±3.8, *n*=3). The [Zn^2+^]_c_ in the BCCAO-operated TRPM2-KO mice appeared to increase compared with the sham-operated TRPM2-KO mice (1.7±0.3, *n*=3), but the increase was not statistically significant (*P*>0.05). We also examined the [Zn^2+^]_c_ in the WT and TRPM2-KO mice at 24 and 48 h, as well as 72 h after BCCAO operation. The increase in the [Zn^2+^]_c_ in the WT mice became discernible at 48–72 h but not at 24 h (Supplementary Figure 5), supporting that TRPM2 deficiency prevents the delayed increase in the [Zn^2+^]_c_.

**Figure 5:**
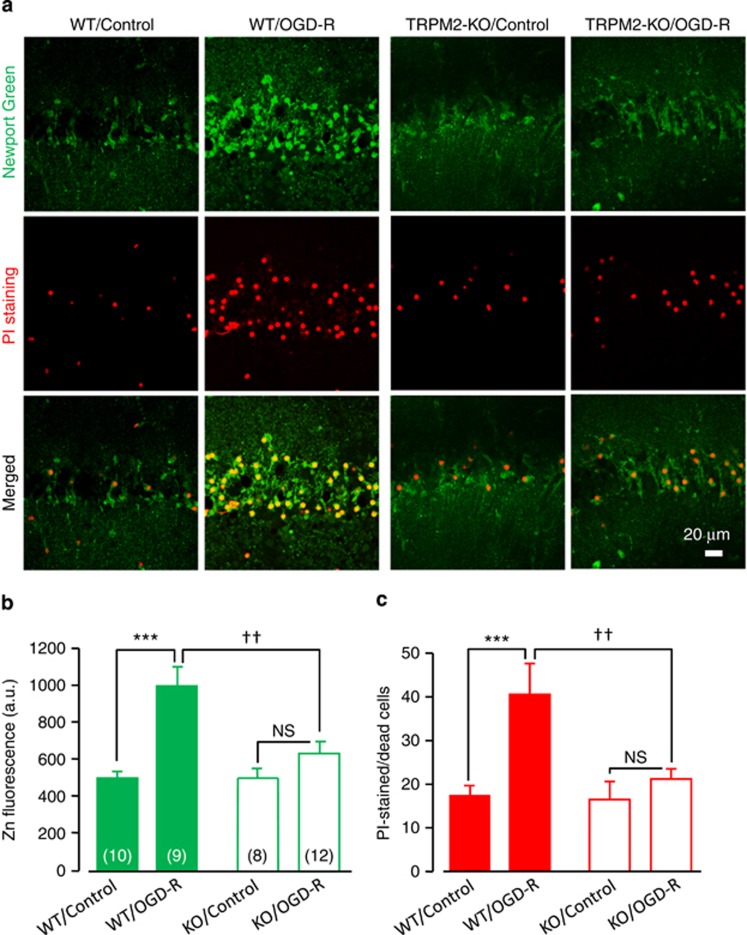
TRPM2 deficiency prevents increases in the [Zn^2+^]_c_ and CA1 pyramidal neuronal death after oxygen glucose deprivation–reperfusion. (**a**) Representative images showing Newport Green and PI staining of WT and TRPM2-KO hippocampal brain slices after OGD-R or control operation. (**b** and **c**) Quantitative analysis of Zn^2+^ fluorescence intensity (**b**) and dead neurons (**c**) as shown in (**a**). The number of slices examined in each case is shown within parentheses in (**c**). ***P<0.005 and NS, no significant difference for comparisons within WT or TRPM2-KO mice; ^††^*P*<0.01 for comparisons between WT and TRPM2-KO mice

To further corroborate these findings, we also used oxygen/glucose deprivation–reperfusion (OGD-R), an *in vitro* model of transient ischemia, in acute hippocampal brain slices (Figure 5a). There were low Zn^2+^ fluorescence and neuronal death in the control slices that were similar between WT and TRPM2-KO mice (Figures 5b and c), likely resulting from slice preparations. OGD-R induced increases in both the [Zn^2+^]_c_ and pyramidal neuronal death in the WT slices as reported previously,^8, 13^ and both responses were absent in the TRPM2-KO slices (Figures 5b and c).

Therefore, our studies using *in vivo* and *in vitro* models of transient ischemia provide consistent evidence to indicate that TRPM2 has an important role in the delayed increase in the [Zn^2+^]_c_ and cell death in CA1 pyramidal neurons. Recent studies have reported that the zinc transporters (ZnT1, ZnT2, ZnT3 and ZnT6) and TRPM7 channel are involved in the regulation of Zn^2+^ homeostasis in the brain.^11, 39, 40^ We performed real-time RT-PCR to test whether TRPM2 deficiency altered their expression. There was no significant difference at the mRNA level in the hippocampus of WT and TRPM2-KO mice (Supplementary Figure 6), suggesting no major role for these Zn^2+^-regulating mechanisms in attenuating the delayed increase in the [Zn^2+^] and the protection against pyramidal neuronal death as a result of TRPM2 deficiency.

### TRPM2 deficiency abolishes reperfusion- and ROS-induced increase in the [Zn^2+^]_c_

**Figure 6:**
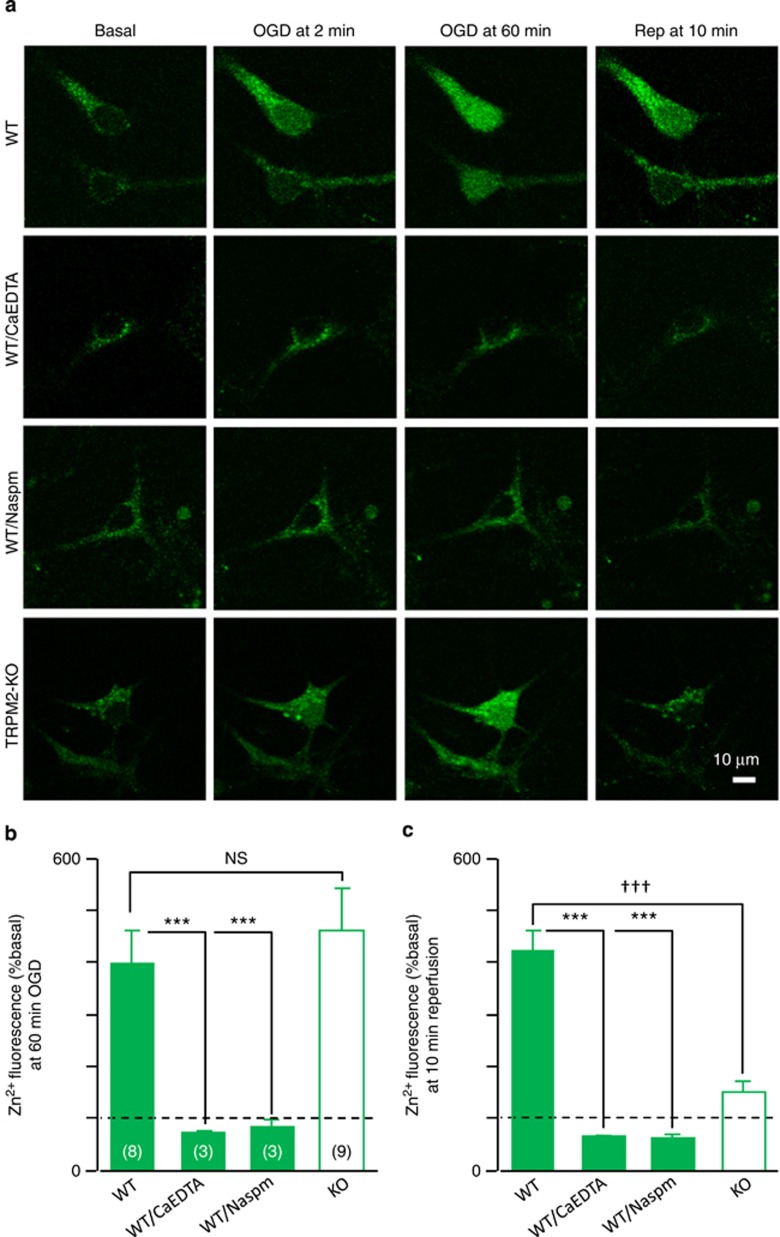
TRPM2 deficiency abolishes the increase in the [Zn^2+^]_c_ during reperfusion without altering Zn^2+^ influx during ischemia in cultured hippocampal neurons. (**a**) Representative images showing the [Zn^2+^]_c_ under basal conditions, 2 and 60 min after OGD and 10 min after reperfusion in cultured hippocampal neurons from WT mice without or with treatment by CaEDTA or Naspm, and from TRPM2-KO mice. (**b** and **c**) Mean changes in the [Zn^2+^]_c_ at 60 min of OGD (**b**) and 10 min of reperfusion (**c**) as shown in (**a**) and expressed as the percentage of the basal [Zn^2+^]_c_ denoted by the dotted lines. The number of neurons examined for each case is shown within parentheses in (**b**). ***P<0.005 for comparisons among different WT groups; ^†††^*P*<0.005; and NS, no significant difference for comparisons between WT and TRPM2-KO mice.

To understand mechanistically how TRPM2 is engaged in the delayed increase in the [Zn^2+^]_c_, we used time-lapse confocal imaging to monitor the temporal changes in the [Zn^2+^]_c_ during OGD-R in cultured hippocampal neurons from the WT and TRPM2-KO mice. OGD induced a robust increase in the [Zn^2+^]_c_ in the WT neurons (Figures 6a and b). Such an increase was completely inhibited using CaEDTA, a membrane-impermeable and specific Zn^2+^ chelator,^14, 15^ or Naspm, a GluR2-lacking AMPAR-selective antagonist^16^ (Figures 6a–c). The TRPM2-KO neurons exhibited similar basal [Zn^2+^]_c_ and OGD-induced increase in the [Zn^2+^]_c_ as the WT neurons (Figures 6a and b). These results confirm a critical role for AMPARs,^13, 14, 16^ and also exclude a role for TRPM2 in elevating the [Zn^2+^]_c_ during ischemia. In stark contrast with the sustained [Zn^2+^]_c_ in the WT neurons upon reperfusion, the [Zn^2+^]_c_ in the TRPM2-KO neurons declined rapidly, returning almost to the basal level in 10 min (Figures 6a and c). Consistent with the well-established fact that reperfusion generates excessive ROS,^41^ our results provide the first evidence to show ROS-sensitive TRPM2 channel as a molecular mechanism that is exclusively required for the delayed increase in the [Zn^2+^]_c_ or cytosolic Zn^2+^ accumulation during reperfusion.

**Figure 7:**
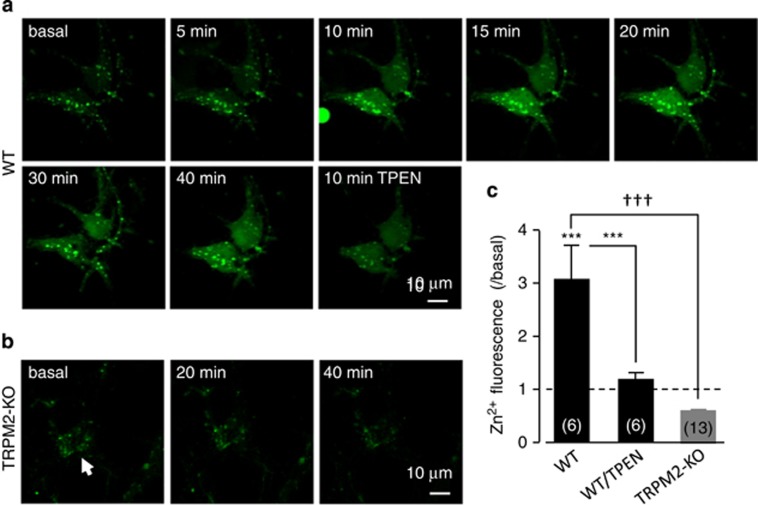
TRPM2 deficiency abolishes H_2_O_2_-induced increase in the [Zn^2+^]_c_ in cultured hippocampal neurons. (**a** and **b**) Representative images showing [Zn^2+^]_c_ in cultured hippocampal neurons from WT and TRPM2-KO mice before (basal) and during exposure to 300 *μ*M H_2_O_2_. The WT neurons were further exposed to 10 *μ*M TPEN for 10 min. The arrow points to the neuron in (**b**). (**c**) Mean [Zn^2+^]_c_ induced by H_2_O_2_ at 40 min and after TPEN treatment as shown in (**a**), and presented relative to the basal [Zn^2+^]_c_ denoted by the dotted line. The number of neurons examined for each case is shown within parentheses in (**c**). ***P<0.005 for comparison with the basal level or before and after TPEN treatment; ^†††^*P*<0.005 for comparison between WT and TRPM2-KO mice

To testify directly whether TRPM2 activation underpins ROS-induced increase in the [Zn^2+^]_c_, we monitored the [Zn^2+^]_c_ in cultured hippocampal neurons during exposure to H_2_O_2_ (Figure 7). H_2_O_2_ at 100–300 *μ*M evoked a salient increase in the [Zn^2+^]_c_, leading to Zn^2+^ accumulation in some punctate structures in the WT neurons as reported previously.^42^ Such H_2_O_2_-induced increase in the [Zn^2+^]_c_ was strongly attenuated by TPEN, a selective Zn^2+^ chelator (Figures 7a and c). More evidently, H_2_O_2_-induced Zn^2+^ responses were completely absent in the TRPM2-KO neurons (Figures 7b and c), further supporting an essential role of TRPM2 in ROS-induced increase in the [Zn^2+^]_c_.

### TRPM2 deficiency attenuates ROS production

**Figure 8:**
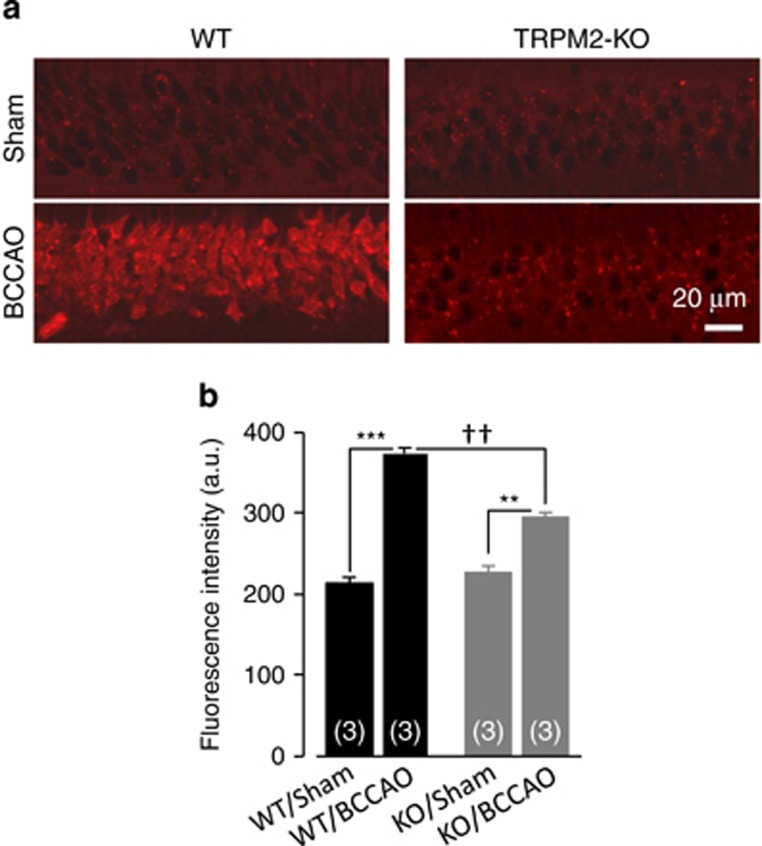
TRPM2 deficiency attenuates ROS generation after transient global ischemia. (**a**) Representative images of dHEt staining of hippocampal slices from BCCAO- or sham-operated WT and TRPM2-KO mice. (**b**) Summary of dHEt staining intensity as shown in (**a**). The number of mice examined for each case is shown within parentheses. **P<0.01 and ***P<0.005 for comparisons within WT or TRPM2-KO mice; ^††^*P*<0.01 for comparisons between WT and TRPM2-KO mice

As mentioned above, Zn^2+^ can activate diverse ROS-generating machineries to generate excessive ROS as oxygen becomes available during reperfusion.^18, 19^ We were interested in whether the loss of TRPM2-dependent increase in the [Zn^2+^]_c_ led to a reduction in ROS production after transient ischemia. Thus, we detected the superoxide production *in situ* in the CA1 region 3.5, 24, 48 and 72 h after BCCAO operation. As expected, in the WT mice, there was a strong increase in ROS production at 3.5 h that was reduced over the next 72 h (Figure 8 and Supplementary Figure 7). The ROS production occurred noticeably earlier than the increases in the [Zn^2+^]_c_ (cf. Supplementary Figure 5). The ROS production in the BCCAO-operated TRPM2-KO mice was also increased, but the ROS level was significantly attenuated in comparison with that detected in the BCCAO-operated TRPM2-KO mice over the same period of examination time (Figure 8 and Supplementary Figure 7). These results indicate that TRPM2 is critical for ROS production during reperfusion.

## Discussion

The present study, using TRPM2-deficient mice in conjunction with *in vivo* and *in vitro* models of transient ischemia, has made several important findings that support a critical role for TRPM2 activation during reperfusion in determining delayed increases in the [Zn^2+^]_c_, CA pyramidal neuronal death, and memory impairments. First, TRPM2 deficiency protected against CA1 pyramidal neuronal death induced by transient ischemia (Figures 2,4 and 5), which are in agreement with recent reports.^27, 28, 29, 30^ Second, preventing transient ischemia-induced CA1 pyramidal neuronal death by TRPM2 deficiency offers strong protection of postischemia memory impairments as assessed by novelty environment habituation test and water maze test (Figure 3), supporting a critical role of TRPM2-dependent CA1 pyramidal neuronal death in the development of learning and memory deficits. Third, TRPM2 was required for the increase in the [Zn^2+^]_c_ (Figure 4) and ROS generation (Figure 8) during reperfusion. Finally, TRPM2 was essential for ROS-induced delayed increase in the [Zn^2+^]_c_ in hippocampal neurons (Figure 7) as well as ROS-induced increase in the [Zn^2+^]_c_ in HEK293 cells (Supplementary Figure 3). Taken together, these findings provide a novel mechanistic insight into the molecular processes underlying transient ischemia-induced brain injury.

It is well established that ischemia–reperfusion leads to the generation of excessive ROS and increases in the [Zn^2+^]_c_, which in turn causes delayed CA1 pyramidal neuronal death and postischemic brain injury.^2, 3, 19, 41^ The underlying Zn^2+^ signaling mechanisms are not fully understood. Presynaptic Zn^2+^ release and subsequent cytosolic accumulation via Zn^2+^-permeable channels and particularly GluR2-lacking AMPARs represent a well-recognized mechanism.^13, 14, 15, 16, 17, 33^ There is accumulating evidence to support synaptic presence of GluR2-lacking AMPARs in CA1 pyramidal neurons,^43, 44, 45, 46, 47, 48^ including a recent study that shows that transient ischemia induces a fast endocytosis of GluA2 subunit and insertion of GluA1 subunit in cultured hippocampal pyramidal neurons.^49^ The finding from the present study that OGD-induced increase in the [Zn^2+^]_c_ was prevented by removing extracellular Zn^2+^ by CaEDTA or by GluA2-lacking AMPAR-specific antagonist Naspm (Figures 6a and b) is highly consistent with the notion of GluA2-lacking AMPARs in hippocampal CA1 pyramidal neurons as the Zn^2+^ influx pathway during ischemia.^13, 14, 15, 16^ TRPM2 deficiency did not alter the basal [Zn^2+^]_c_ and ischemia-induced increase in the [Zn^2+^]_c_, strongly excluding a role for TRPM2 in mediating ischemia-induced in the [Zn^2+^]_c_ (Figure 6b). In striking contrast, TRPM2 deficiency caused rapid loss of the cytosolic Zn^2+^ upon reperfusion (Figure 6c), revealing an exclusive role for the TRPM2 activation during reperfusion in the delayed increases in the [Zn^2+^]_c_ (Figure 4 and Supplementary Figure 6). This finding is well reconciled with the facts that reperfusion provides oxygen, which the ROS-generating machineries need to generate ROS during reperfusion^37^ (Figure 8 and Supplementary Figure 7), and that TRPM2 is an important ROS sensor^22, 23, 24, 25^ and activation of TRPM2 channels leads to increases in the [Zn^2+^]_c_.^30^ Furthermore, consistent with the notion that Zn^2+^ can induce further ROS production *via* activating various ROS-generating mechanisms,^8, 13, 19, 20, 21, 39, 47^ TRPM2 deficiency strongly but incompletely suppressed ROS generation (Figure 8). Selective loss of cytosolic Zn^2+^ accumulation as a result of TRPM2 deficiency also attenuated ROS production during reperfusion (Supplementary Figure 7) and potent neuronal protection (Figures 2, 3, 4, 5), and provided compelling evidence to suggest TRPM2 activation during reperfusion is a key step initiating a vicious positive feedback mechanism driving the delayed increase in the [Zn^2+^]_c_ and cell death in CA1 pyramidal neurons and memory impairments after transient ischemia.

Such an exclusive role of TRPM2 during reperfusion predicts that TRPM2 deficiency can only protect against brain damage induced by transient (followed by reperfusion) but not permanent (no reperfusion) ischemia, which has been shown in a recent study.^29^ The postischemic TRPM2-dependent Zn^2+^ mechanism revealed in the present study can also explain satisfactorily why removal of Zn^2+^ by CaEGTA^15^ or pharmacological inhibition of TRPM2^27^ after transient ischemia was also effective in preventing the delayed CA1 pyramidal neuronal death.^15, 27^ The present study shows that TRPM2 is present on hippocampal pyramidal neurons (Figure 1), but whether it also has intracellular localization, as found in other cell types,^50^ remains to be investigated. Further studies are required to determine the sources responsible for the increase in the [Zn^2+^]_c_ during reperfusion and the mechanisms responsible for further ROS production. H_2_O_2_-induced increase in the [Zn^2+^]_c_ in cultured hippocampal neurons was observed in extracellular Zn^2+^-free solutions (Figure 7) and, as suggested previously,^51^ may result at least in part from intracellular Zn^2+^ release. It should be mentioned that TRPM2 is expressed in glial cells as well as in neurons in the brain;^23^ non-neuronal TRPM2 may also be involved in neuronal death induced by transient ischemia, and studies are required to study their contribution.

In summary, the present study shows TRPM2 activation during reperfusion as a crucial mechanism responsible for the delayed increases in the [Zn^2+^]_c_ that drives transient ischemia-induced CA1 pyramidal neuronal death and memory impairments, and suggests that TRPM2 inhibition is a promising strategy of developing novel therapeutic treatments to mitigate the cognitive sequelae following transient ischemia or stroke.

## Materials and Methods

### Mice

TRPM2-KO mice were generated in the C57BL/6 background as detailed in our recent study.^52^ WT and TRPM2-KO mice were housed under standard conditions with a 12/12 h light/dark cycle and free access to food and water. All animal use procedures were approved by the Committees at Zhejiang University and Leeds University for the Care and Use of Laboratory Animals. All the experiments were performed at room temperature or specifically indicated. The behavioral studies and the analysis of images were performed double-blindly without the knowledge of the treatments given to the animals.

### BCCAO and reperfusion

The bilateral common carotid arteries in 8- to 12-week-old male mice were disclosed by incision and occluded using microaneurysm clips for 15 min under 2% isoflurane in a mix gas of 70% N_2_O/30% O_2_ using a face mask. The body temperature was maintained at 37±0.5 °C by a heating pad during surgery by monitoring rectal temperature with a digital thermometer. After blood flow was restored and incision sutured, the mice were kept at 37±2 °C for ≥4 h and recovered under normal housing conditions for the indicated time. The cerebral blood flow was monitored by the laser Doppler flowmetry (Moor, Devon, UK) to confirm the ceasing and restoration of blood flow. The survival rate was calculated at 12, 24, 48 and 72 h after BCCAO operation.

### Hippocampal neuron culture

Hippocampal tissues from postnatal day 1 mice were chopped and digested in 0.5% trypsin in Hank's balanced salt solution (HBSS) at 37 °C for 13 min. Dissociated cells were plated at 1 × 10^5^/cm^2^ in poly-L-lysine-coated 35-mm dishes and incubated in the Neurobasal medium containing 0.5 mM glutamax, 50 U/ml penicillin, 50 *μ*g/ml streptomycin and 2% B27 (Invitrogen, Carlsbad, CA, USA) for 3 days. Cells were maintained in the Neurobasal medium containing 2.5 *μ*M cytosine arabinofuranoside, which was replaced every 4 days.

### Immunostaining, Nissl and TSQ staining

Mice were anesthetized with sodium pentobarbital and killed at 24, 48 and 72 h after BCCAO, respectively. For immunostaining, brains were fixed with 4% paraformaldehyde and cut into 30-*μ*m-thick coronal sections (1.58–2.18 mm posterior to Bregma) using a Leica CM3050S cryostat (Leica Biosystems, Mannheim, Germany). After blocking with goat serum in phosphate-buffered saline (PBS) containing 0.2% Triton X-100 for 1 h, slices were incubated with rabbit anti-TRPM2 (Abcam, Cambridge, UK; 1 : 200), mouse anti-CaMKII (Abcam; 1 : 500), mouse anti-GFAP (Invitrogen; 1 : 200) or guinea-pig anti-NeuN (Millipore, Billerica, MA, USA; 1 : 1000) at 4 °C overnight, and then with Alexa Flour 546 anti-rabbit IgG, Alexa Flour 546 anti-guinea-pig IgG or Alexa Flour 488 anti-mouse IgG antibody (Invitrogen; 1 : 1000) for 1 h, followed with DAPI staining (Invitrogen; 1 : 10 000) for 15 min in some experiments. Nissl staining was performed according to the manufacturer's instructions (Beyotime, Suzhou, China). For TSQ and Nissl staining, brains were frozen and cut into 20-*μ*m-thick coronal sections. Air-dried slices were stained with 100 *μ*M TSQ (Molecular Probes, Invitrogen) for 90 s and, after the fluorescent images were captured, stained with Nissl. Images were captured using an Olympus FV1000 confocal microscope (Olympus, Tokyo, Japan) and Image-Pro Plus (Media Cybernetics, Inc., Bethesda, MD, USA).

For immunostaining of single cultured hippocampal neurons, cells were seeded at a density of ~65 cells/mm^2^ on 13-mm poly-L-Lysine-coated coverslips placed in 24-well plates and cultured for 21 days *in vitro* (DIV). Cells were incubated in fixing solution (PBS containing 4% paraformaldehyde and 4% sucrose) for 10 min. After washing in PBS, cells were incubated with blocking serum solution (PBS containing 5% BSA and 0.4% Triton X-100) for 1 h at room temperature. Cells were incubated with rabbit anti-TRPM2 antibody (Bethyl, Montgomery, TX, USA; 1 : 1000) and mouse anti-Neuro-ChromTM Pan Neuronal Marker antibody (Millipore; 1 : 100) in blocking serum solution at 4 °C overnight and, after washing in PBS, incubated with fluorescein isothiocyanate-conjugated anti-rabbit IgG secondary antibody (Sigma, St. Louis, MO, USA; 1 : 600) for 1 h. Cells were washed with PBS and incubated with tertramethylrhodamine isothiocyanate-conjugated anti-mouse IgG secondary antibody (Sigma; 1 : 500) for 1 h. After cells were washed with PBS and rinsed in water, coverslips were mounted with SlowFade Gold antifade reagent with DAPI (Invitrogen) and kept in 4 °C overnight. Fluorescent images were captured using an inverted LSM700 microscope (Carl Zeiss, Jena, Germany).

### Acute brain slice preparation

Four-week-old male mice were anesthetized with diethyl ether and brains were cut into 300-*μ*m-thick transverse slices using a Leica 1200S vibrotome (Mannheim, Germany) in ice-cold solutions (in mM: 2.5 KCl, 7 MgSO_4_, 0.5 CaCl_2_, 1.25 NaH_2_PO_4_, 25 NaHCO_3_, 10 glucose and 210 sucrose, pH 7.3). Slices were firstly incubated at 34 °C to recover for 30 min and then at room temperature for ≥45 min in artificial cerebral spinal fluid (ACSF) (in mM: 119 NaCl, 2.5 KCl, 1 NaH_2_PO_4_, 26.2 NaHCO_3_, 2.5 CaCl_2_, 1.3 MgSO_4_ and 11 glucose, pH 7.3) bubbled with 95% O_2_/5% CO_2_.

### Patch-clamp recording

Whole-cell currents were recorded from cultured hippocampal neurons or acute brain slices, using an Axopatch 200B or Multiclamp 700B amplifier (Molecular Devices, Sunnyvale, CA, USA) and protocols as described previously.^32^ Intracellular solution for recording cultured neurons contained (in mM): 147 Cs-gluconate, 10 HEPES, 2 MgCl_2_ and 1 ADPR (pH 7.3) and extracellular solution: 147 NaCl, 2 KCl, 10 HEPES, 13 glucose, 2 CaCl_2_ and 0.2 *μ*M TTX (pH 7.4). Intracellular solution for recording brain slices contained (in mM): 135 CsMeSO_4_, 8 NaCl, 10 HEPES, 4 MgATP, 0.3 EGTA, 0.3 ADPR and 5 QX314 (pH 7.3), and Mg^2+^-free ACSF was used as an extracellular solution. Clotrimazol at 20 *μ*M was diluted in an extracellular solution. The signals were filtered at 2 kHz and digitized at 10 kHz.

### Open-field test

The open-field test was conducted in an open plastic chamber (45 cm × 45 cm × 45 cm). Mice were individually placed in the corner of the chamber and allowed to freely explore for 15 min. The locomotor activity of the animals in the field was measured using an automated video-tracking system (ViewPoint, Lyon, France). Measurement began immediately after placement in the chamber. The total distance mice traveled was calculated, and the accumulative time mice spent in the central square of the open plastic chamber was used as an indicator for exploratory ability.

### Novelty environment habituation test

Similar to the open-field test, the mice were placed in an open plastic chamber (45 cm × 45 cm × 45 cm) and allowed to move freely for 5 min, and their locomotor activities were monitored using a ViewPoint video-tracking system. This test was repeated further two times to examine their memory of the pre-exposed chamber environments, and the mice were subjected to sham or BCCAO operation after the second test. The novelty environment habituation score was presented as the locomotion activity during the second and third tests as the percentage of that during the first test.

### Water maze test

This spatial learning and memory test was conducted as described in a previous study.^53^ In brief, the circular water maze tank of 100 cm in diameter and 60 cm deep was filled with water and the water temperature was maintained at 22±1 °C. The water was opaque by adding white and non-toxic paint. A 10-cm diameter escape platform was submerged 0.5 cm below the water surface in a fixed position. Distinct cues were painted on the walls. The whole procedure was performed over a period of 6 days, composed of a training session during the first 5 days and the probe test on the last day. Each mouse, 7 days after sham or BCCAO operation, was trained four trails with a 30- s intertrial interval for each day during the training session. The mouse was placed in one of the four random start locations facing the wall. The trial was complete when the mouse touched the platform or 60 s elapsed. If the mouse failed to find the platform with the limited time during a given trial, it was moved onto the platform by the experimenter. The probe test lasted for 60 s and was conducted 24 h after the last training using the same water tank with the escape platform removed. The latency for mice to reach the escape platform during the training and the time mice spent searching for the missing escape platform in the target quadrant during the probe test were recorded and analyzed using an automated tracking system (Coulbourn Instrument, Whitehall, PA, USA; ACTIMETRICS software, Wilmette, IL, USA).

### *In situ* detection of ROS production

The production of superoxide *in situ* was detected as follows. Dihydroethidium (dHEt) (Invitrogen) at 1 mg/kg mouse weight was injected intraperitoneally at the beginning of BCCAO. Mice were killed 3.5, 24, 48 and 72 h after termination of BCCAO and reperfusion and fixed with 4% PFA in PBS, respectively. The brain was fixed further in 4% PFA overnight. Three hippocampal sections with 150 *μ*m intervals were prepared (Vibrotome 1000 plus, Royston, Herts, UK). The image was captured by a confocal fluorescent microscope with excitation at 510–550 nm and emission at 580 nm. Ethidium signal intensity was expressed as the mean fluorescence in neuronal body in the hippocampal CA1 region. Data were obtained from three independent experiments.

### OGD and reperfusion

Glucose-free ACSF with glucose replaced with NaCl and normal ACSF were bubbled, 30 min before and during use, with 95% N_2_/5% CO_2_ or 95% O_2_/5% CO_2_, respectively. Acute brain slices were perfused in normal ACSF for 30 min, glucose-free ACSF for 1 h and then washed with normal ACSF for 30 min, whereas the control slices remained in normal ACSF for 2 h. The slices were then incubated for 10 min in normal ACSF containing 5 *μ*g/ml propidium iodide (PI) and 20 *μ*M Newport Green (Invitrogen). Fluorescence images were captured using an Olympus FV1000 confocal microscope and Zn^2+^ fluorescence intensity was measured using ImageJ software (NIH, Bethesda, MD, USA), and PI-stained cells were counted in the same stratum pyramidal layer of the CA1 region. Data were from at least three independent experiments.

### Cell imaging

Hippocampal neurons of 12–14 DIV were incubated with 1 *μ*M FluoZin3-AM and 0.02% F127 (Invitrogen) at 37 °C for 1 h and maintained for another hour in normal solutions (in mM:129 NaCl, 5 KCl, 3 CaCl_2_, 1 MgCl_2_, 10 glucose and 10 HEPES, pH 7.4). Neurons were perfused for 60 min with glucose-free solution (in mM: 140 NaCl, 5 KCl, 3 CaCl_2_, 0.05 glycine and 10 HEPES, pH 7.4) saturated with 95% N_2_/5% CO_2_ and then normal solution for 10 min. In some experiments, 100 *μ*M CaEDTA or 25 *μ*M Naspm was included. DIV14 hippocampal neurons used to study H_2_O_2_-induced changes in the [Zn^2+^]_c_ were loaded with 1 *μ*M FluoZin3-AM in a similar manner in HBSS. Neurons were exposed to H_2_O_2_ at 100 or 300 *μ*M for 40 min, followed by treatment with 10 *μ*M TPEN for 10 min in some experiments. Fluorescence images were captured using an Olympus FV1000 confocal microscope or a Zeiss M510 confocal microscope (Carl Zeiss) and fluorescence intensity was measured using ImageJ software. Data were from at least three independent experiments.

Single-cell imaging of Zn^2+^ influence in a Tet-inducible HEK293 cells conditionally expressing TRPM2 was carried out as described above for cultured hippocampal neurons using extracellular solutions (in mM: 147 NaCl, 2 KCl, 2 CaCl_2_, 1 MgCl_2_, 13 glucose and 10 HEPES, pH 7.4) that contained no or 10 *μ*M ZnCl_2_. The cells were maintained in DMEM/F-12 (Invitrogen) supplemented with 10% fetal bovine serum and GlutaMaxTM-1 (Gibco, Life Technologies, Paisley, UK), 5 *μ*g/ml blasticidin (Invitrogen) and 400 *μ*g/ml zeocin (Invitrogen). Expression of TRPM2 was induced by removing blasticidin and zeocin and adding 1 *μ*g/ml Tet in the culture medium. Cells were seeded onto 35-mm glass-bottomed dishes (FluoroDish; World Precision Instruments, Sarasota, FL, USA) and grown to ~60% confluency. Total fluorescence intensity was measured with ImageJ software. Data were from at least three independent experiments.

### Real-time RT-PCR

Total RNA was extracted from 20–30  mg of the hippocampus from each mouse using the RNeasy Mini-prep Kit according to the manufacturer's instructions (Takara, Dalian, China). Real-time RT-PCR analysis was performed in four duplicates for each sample using the QPCR SYBR Green Fluorescein Mix (Takara) and a CFX-96 Thermal Cycler (Bio-Rad, Hercules, CA, USA). In all, 500–2000 ng of mRNA was reverse-transcribed using the OmniScript Kit (Takara). Each PCR reaction contained 2 *μ*l of diluted (1 : 10) cDNA, primers (0.4 *μ*M each) and dNTPs (0.35 *μ*M each). The PCR protocols were composed of one initial step of 95 °C for 2 min, 40 cycles of 95 °C for 30 s, 60 °C for 30 s and 72 °C for 30 s, and one additional step of 72 °C for 10 min. The forward and reverse primer sequences used were as follows: 5′-TACTGGGCACAGTGAATGG-3′ and 5′-GCAAGGCTAAGGAGAAGACC-3′ for ZnT1; 5′-ATGCTCATTAGCCTCTTCGC-3′ and 5′-CTGTCGTCACGGCTGTTCC-3′ for ZnT2; 5′-TTCCACCACTGCCACAAG-3′ and 5′-TGCTAAATACCCACCAACCA-3′ for ZnT3; 5′-TCCTCCAGACAACACCACC-3′ and 5′-AGCCAATGAGCCAAATCC-3′ for ZnT6; 5′-CAGGCTATGCTTGATGCTCT-3′ and 5′-GGTTGGACCTTGTTTAGTGTTAT-3′ for TRPM7; and 5′-AGAGTGTTTCCTCGTCCCG-3′ and 5′-CCGTTGAATTTGCCGTGA-3′ for glyceraldehyde- 3-phosphate dehydrogenase (GAPDH). The intensity relative to GAPDH was estimated by the ΔΔC_T_ method. The size of PCR products was confirmed by agarose gel analysis. The data from four duplicates were averaged, and the average value from the TRPM2-KO mice was normalized to that in the matched WT mice in each independent experiment before the mean value was obtained from all the mice examined.

### Data analysis

All data are presented with mean±S.E.M. where appropriate. Damaged neurons identified by condensed cell body and nuclei in Nissl staining, NeuN-positive neurons and GFAP-positive glial cells were counted within an area of 0.2 mm^2^ randomly chosen for three adjacent fields. The TSQ intensity in CA1 pyramidal neurons was normalized to that in lateral ventricles. The changes in the [Zn^2+^]_c_ in individual neurons and HEK293 cells were expressed relative to the basal level. Statistical analysis was performed using Student's *t*-test or one-way ANOVA followed by *post hoc* Tukey's test, with *P*<0.05 being considered statistically significant.

## Abbreviations

TRPM2: transient receptor potential melastatin-related 2
[Zn^2+^]_c_: cytosolic Zn^2+^ level
ROS: reactive oxygen species
AMPA: α-amino-3-hydroxy-5-methyl-4-isoxazolepropionate
AMPAR: AMPA receptor
ADPR: ADP-ribose
NMDA: *N*-methyl-*D*-aspartate
WT: wild type
CaMKII: calmodulin-dependent kinase II
KO: knockout
GFAP: glial fibrillary acidic protein
BCCAO: bilateral common carotid artery occlusion
CLT: clotrimazole
Tet: tetracycline
HEK293: human embryonic kidney 293
TSQ: 6-methoxy-(8-*p*-toluenesulfonamido)quinolone
OGD-R: oxygen/glucose deprivation reperfusion
ZnT: zinc transporter
TRPM7: transient receptor potential melastatin-related 7
CaEGTA: Ca^2+^ saturated ethylene glycol tetraacetic acid
TPEN: *N,N,N',N'*-tetrakis(2-pyridylmethyl)ethylenediamine
HBSS: Hank's balanced salt solution
PBS: phosphate-buffered saline
DIV: days *in vitro*
DAPI: 4',6-diamidino-2-phenylindole
ACSF: artificial cerebral spinal fluid
dHEt: dihydroethidium
DMEM: Dulbecco's modified Eagle's medium
GAPDH: glyceraldehyde-3-phosphatedehydrogenase
PCR: polymerase chain reaction
