# Supplementary material for: TRPM2 channel deficiency prevents delayed cytosolic Zn2+ accumulation and CA1 pyramidal neuronal death after transient global ischemia
Source: Cell Death Dis. 2014 Nov 27;5(11):e1541–. doi: 10.1038/cddis.2014.494 (PMC4260752; doi:10.1038/cddis.2014.494)

Supplemental data

TRPM2 channel deficiency prevents delayed cytosolic Zn^{2+} accumulation and CA1 pyramidal neuronal death after transient global ischemia

Mao Ye[1*], Wei Yang[1, 2*†], Justin F Ainscough[3], Xupang Hu[1], Xin Li[2], Alicia Sedo[2], Xinheng Zhang[1], Xiangnan Zhang[1], Zhong Chen[1], Xiaoming Li[1], David J Beech[2,3], Asipu Sivaprasadarao[2], Jianhong Luo[1†], Lin-Hua Jiang[2†]

[1]Institute of Neuroscience, Key Laboratory of Medical Neurobiology of the Ministry of Health of China, Zhejiang Province Key Laboratory of Neurobiology, Zhejiang University School of Medicine, Hangzhou, Zhejiang 310058, China; [2]School of Biomedical Sciences, Faculty of Biological Sciences, and [3]School of Medicine, Faculty of Health and Medicine, University of Leeds, Leeds LS2 9JT, United Kingdom

Figure 1 Expression of TRPM2 in cultured hippocampal neurons. Representative images showing staining of cultured hippocampal neurons from WT mice with anti-TRPM2 (green color), a neuronal marker (red color), DAPI (blue color) and the merged image.

Figure 2 TRPM2 deficiency causes no change in neurons and glial cells. (a) Representative images of NeuN staining of hippocampal slices from WT and

TRPM2-KO mice at P7, P21 and adult stage. (**b**) Representative images of GFAP (green color) and DAPI (blue color) staining of hippocampal slices from adult WT and TRPM2-KO mice. (**c**) Summary of the number of GFAP positive cells per $0.2mm^2$ in hippocampus for WT or TRPM2-KO mice as shown in (**b**). (**d**) Summary of the body weight of WT or TRPM2-KO mice. The number of mice was examined in each cases is shown in parenthesis.

Figure 3 TRPM2 deficiency causes no change in locomotor activity. (a) Representative tracking traces showing the locomotor activity of WT mice and TRPM2-KO mice during 15-min open field test. **(b)** Summary of the total distances for the WT or TRPM2-KO mice during indicated period of times. **(c)** Summary of the average time in the central area for the WT or TRPM2-KO mice during indicated period of times. The number of mice was examined in each cases is shown in parenthesis in (**b**).

Figure 4 Expression of TRPM2 confers H_2O_2-induced increases in the $[Zn^{2+}]_c$ in HEK293 cells. (**a**) Representative confocal fluorescent images showing the $[Zn^{2+}]_c$ induced by 300μM H_2O_2 in a tetracycline-induced HEK293 (cell Tet^+) expressing TRPM2 channels (top), and in a un-induced cell lack of TRPM2 expression (bottom). The cells were initially in extracellular Zn^{2+}-free solution and then in extracellular solutions containing 10 μM $ZnCl_2$. The TRPM2-expresing cell was treated with 10 mM TPEN at the end of experiment. (**b**) Summary of Zn^{2+} fluorescent intensity in

Tet^+ cells or Tet^- cells as shown in (**a**). The number of cells examined for each case is shown in parenthesis.

Figure 5 The increase in the $[Zn^{2+}]_c$ in hippocampal neurons after transient global ischemia. Representative images showing TSQ staining of hippocampal slices from the WT and TRPM2-KO mice at 24, 48 and 72 hrs after BCCAO operation. The arrows point to the stratum pyramidal layers where there was a discernible increase in the $[Zn^{2+}]_c$ at 48 and 72 hrs in the WT mice, which were absent in the TRPM2-KO mice.

Figure 6 No change in mRNA expression of zinc transporters and TRPM7. Real-time RT-PCR analysis shows no significant difference the mRNA expression level of ZnT1, ZnT2, ZnT3 and ZnT6 **(a),** and TRPM7 (**b**) in hippocampus of the WT mice and TRPM2-KO mice. The data from the TRPM2-KO mice is normalized to that from the WT mice. The mean data were obtained from six individual WT or TRPM2-KO mice.

Figure 7 ROS generation in hippocampal neurons after transient global ischemia. Representative images of dHEt staining of hippocampal slices from the WT and TRPM2-KO mice at 24, 48 and 72 hrs after BCCAO operation. There was less ROS production in the TRPM2-KO mice.

Supplemental Fig. 1

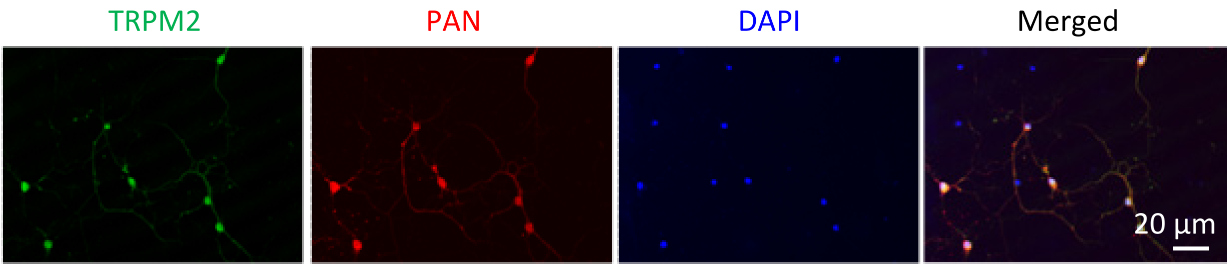

Supplemental Fig. 2

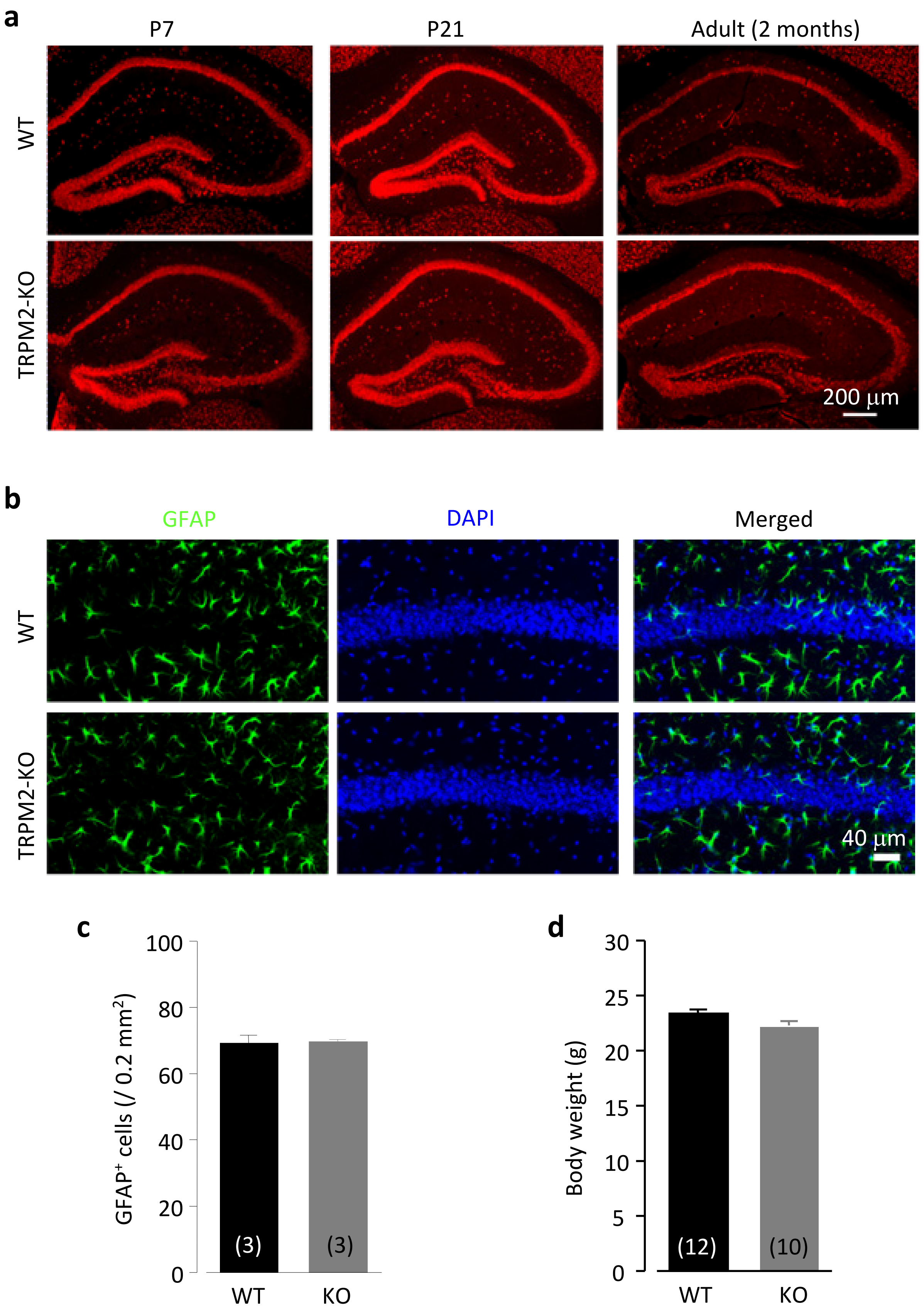

Supplemental Fig. 3

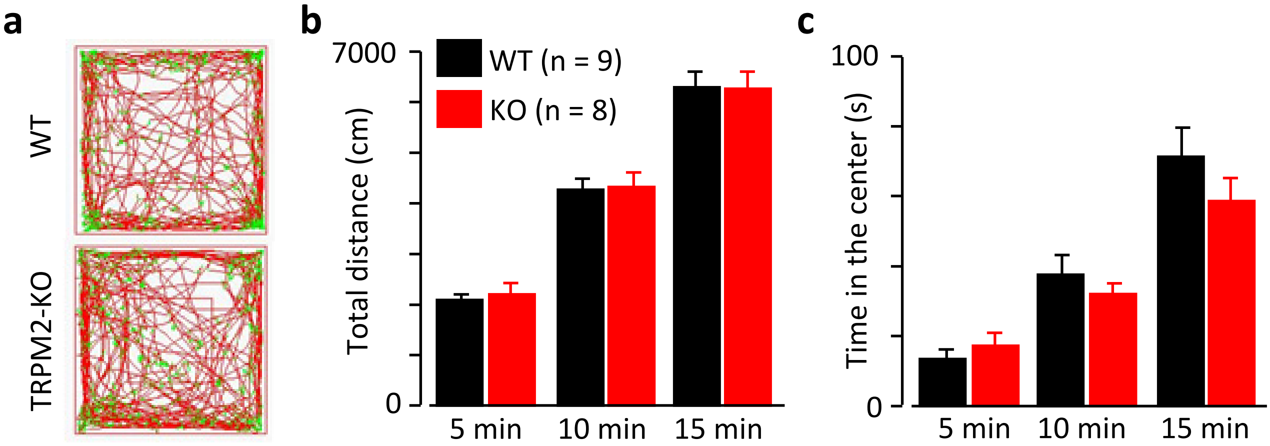

Supplemental Fig. 4

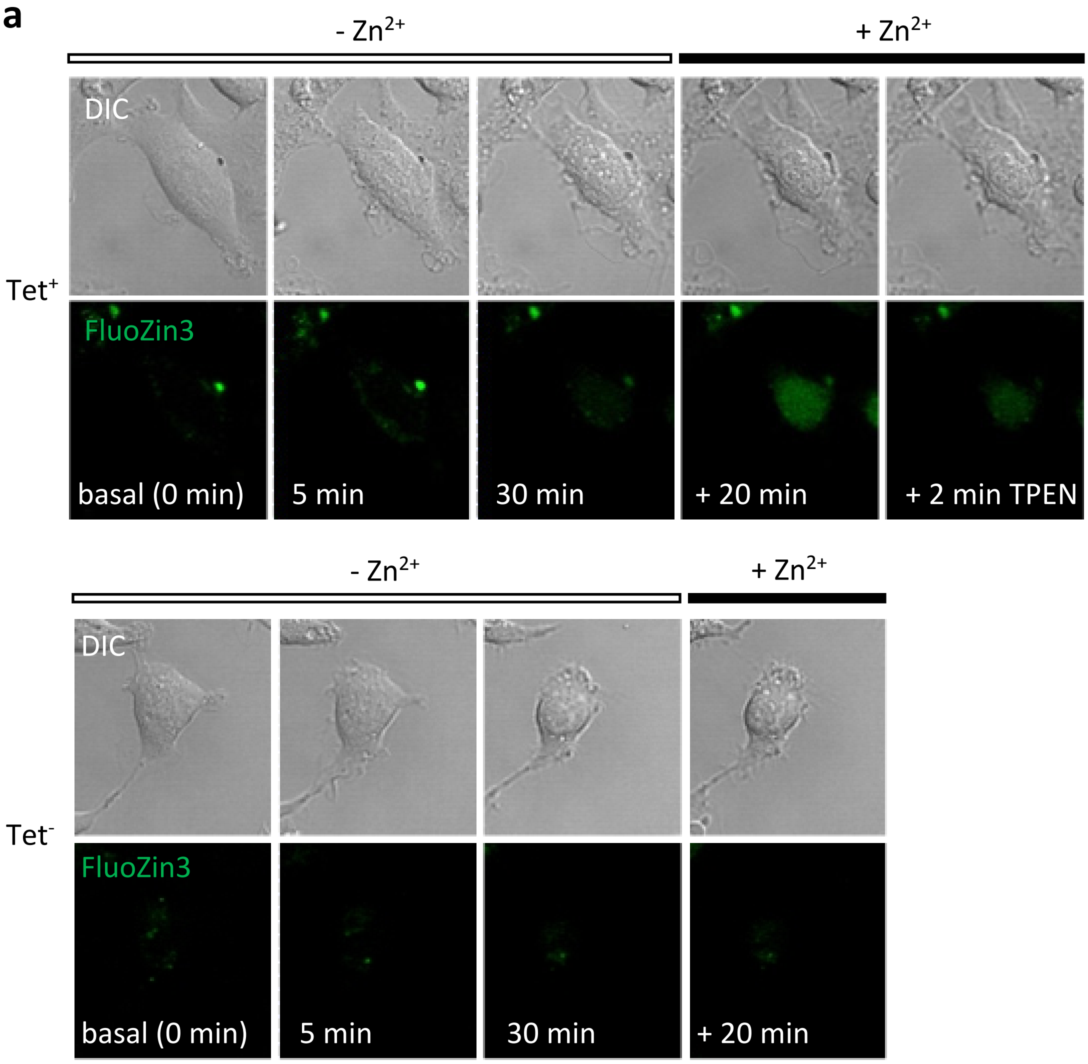

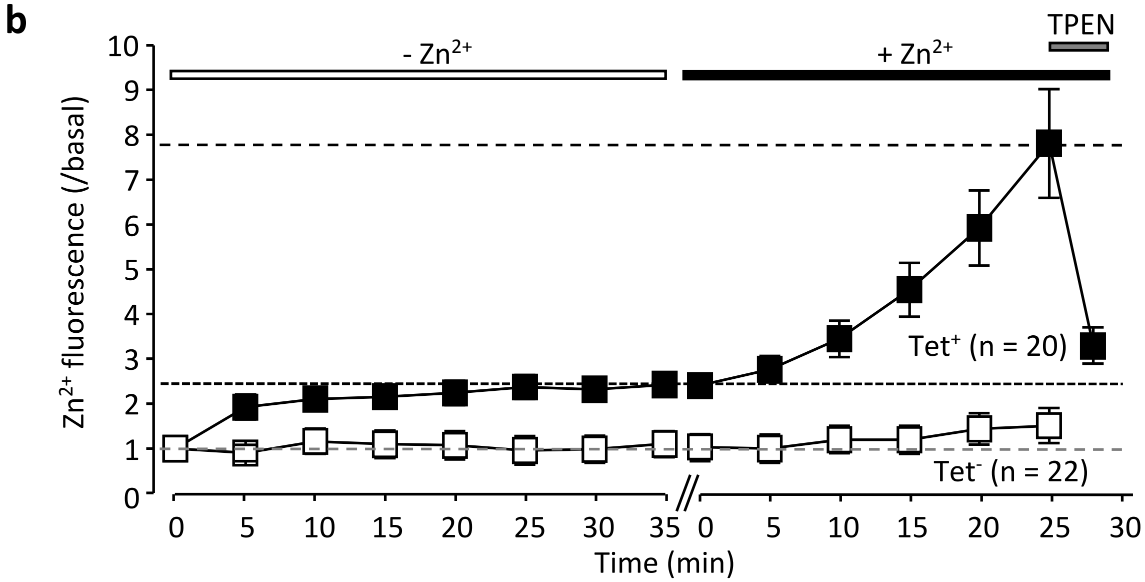

Supplemental Fig. 5

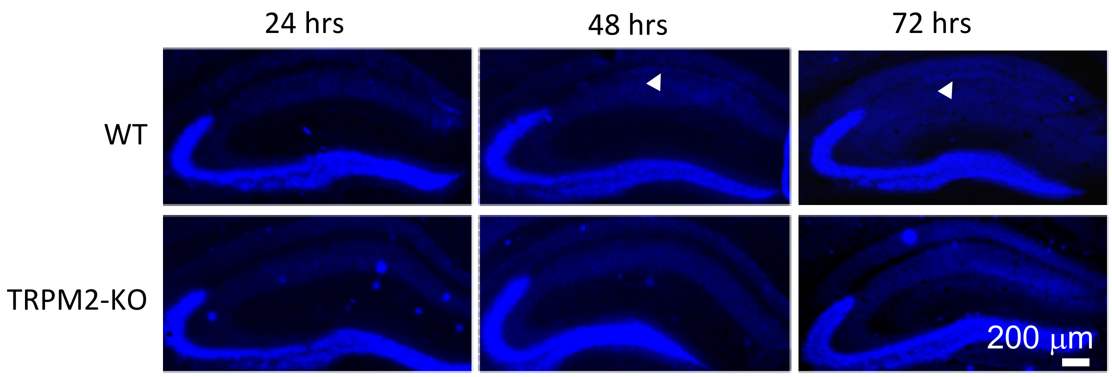

Supplemental Fig. 6

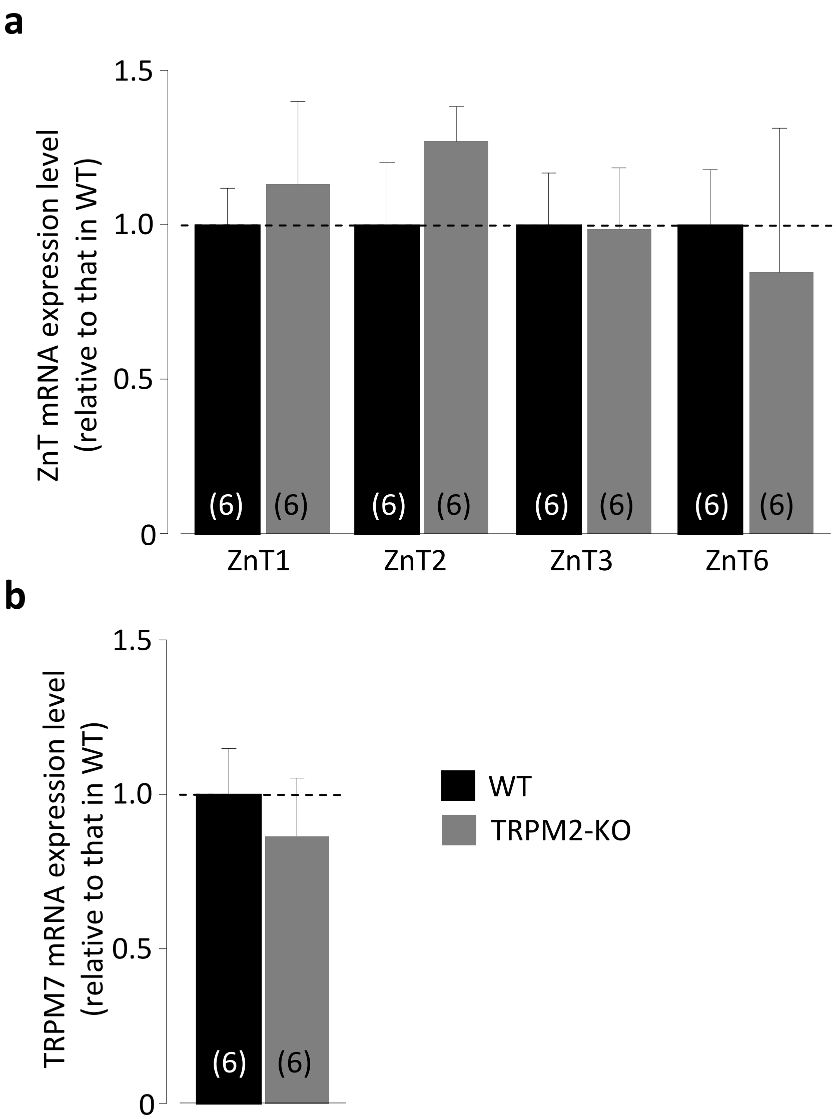

Supplemental Fig. 7

a

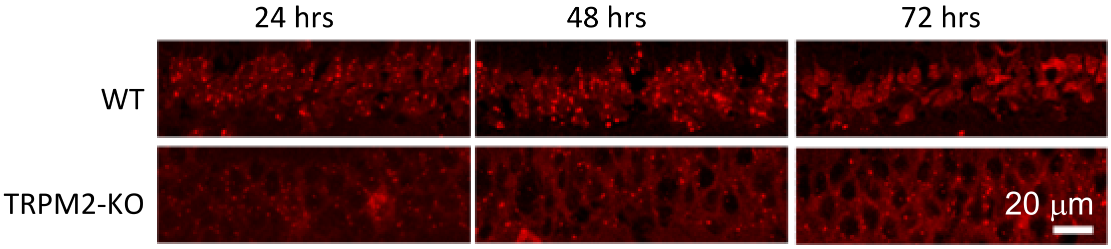

Supplement: Supplementary Information [file cddis2014494x1.pdf]
